# Identifying Environmental Determinants Relevant to Health and Wellbeing in Remote Australian Indigenous Communities: A Scoping Review of Grey Literature

**DOI:** 10.3390/ijerph18084167

**Published:** 2021-04-15

**Authors:** Amal Chakraborty, Mark Daniel, Natasha J. Howard, Alwin Chong, Nicola Slavin, Alex Brown, Margaret Cargo

**Affiliations:** 1 School of Health Sciences, University of South Australia, Adelaide, SA 5001, Australia; mark.daniel@canberra.edu.au (M.D.); natasha.howard@sahmri.com (N.J.H.); alex.brown@sahmri.com (A.B.); margaret.cargo@canberra.edu.au (M.C.); 2 Research Centre for Palliative Care, Death and Dying, College of Nursing and Health Sciences, Flinders University, Bedford Park, SA 5042, Australia; 3 Health Research Institute, Faculty of Health, University of Canberra, Bruce, ACT 2601, Australia; 4 South Australian Health and Medical Research Institute, Adelaide, SA 5000, Australia; 5 Wardliparingga Aboriginal Health Equity, South Australian Health and Medical Research Institute, Adelaide, SA 5000, Australia; 6 Adelaide Medical School, Faculty of Health and Medical Sciences, University of Adelaide, Adelaide, SA 5000, Australia; 7 Australian Centre for Child Protection, University of South Australia, Adelaide, SA 5001, Australia; marawuy@bigpond.com; 8 Environmental Health Branch, Department of Health, Northern Territory Government, Casuarina, NT 0810, Australia; nicola.slavin@nt.gov.au

**Keywords:** indigenous, public health, environmental health, built environment, social planning, public policy, community infrastructure, community capacity, environmental indicators, grey literature

## Abstract

The high prevalence of preventable infectious and chronic diseases in Australian Indigenous populations is a major public health concern. Existing research has rarely examined the role of built and socio-political environmental factors relating to remote Indigenous health and wellbeing. This research identified built and socio-political environmental indicators from publicly available grey literature documents locally-relevant to remote Indigenous communities in the Northern Territory (NT), Australia. Existing planning documents with evidence of community input were used to reduce the response burden on Indigenous communities. A scoping review of community-focused planning documents resulted in the identification of 1120 built and 2215 socio-political environmental indicators. Indicators were systematically classified using an Indigenous indicator classification system (IICS). Applying the IICS yielded indicators prominently featuring the “community infrastructure” domain within the built environment, and the “community capacity” domain within the socio-political environment. This research demonstrates the utility of utilizing existing planning documents and a culturally appropriate systematic classification system to consolidate environmental determinants that influence health and disease occurrence. The findings also support understanding of which features of community-level built and socio-political environments amenable to public health and social policy actions might be targeted to help reduce the prevalence of infectious and chronic diseases in Indigenous communities.

## 1. Introduction

The quality of the places where people live and the opportunities places provide for making healthy choices shape people’s health behavior and their risk factors for health and disease [1]. The World Health Organization (WHO) estimates about 22% of the global burden of disease, and 23% of all deaths are attributable to modifiable environmental factors [2]. Built and social environmental factors are key contributors to health and wellbeing via different biological, behavioral, and psychosocial pathways in Indigenous populations [3,4]. For example, the features of local community contexts (e.g., conditions of living, health enabling resource availability) can directly influence the life course of an individual and lead to variations in people’s behavioral practices, psychosocial factors, stress axes, and inflammation [4]. Despite Australia being ranked second on the global Human Development Index preceded by Norway [5], Aboriginal and Torres Strait Islander Australians (hereafter respectfully referred to as “Indigenous Australians”) have the lowest life expectancy when compared with Indigenous populations in other developed nations such as Canada, USA, and New Zealand [6]. Causes of this disparity are complex, but the continuing effect of colonization, social and political oppression, and dispossession of lands and resources to-date have contributed to significant health and socio-economic inequities implicating substantial health indices in Australian Indigenous populations [7].

Indigenous Australians living in remote areas experience a greater burden of disease and higher mortality rates compared to Indigenous people living in rural and metropolitan areas [8]. Moreover, Indigenous people living in remote and very remote areas of the Northern Territory (NT) are disproportionately disadvantaged compared to all other Australian states and territories [9,10,11]. The gaps in life expectancy, mortality, and disease burden between Indigenous and non-Indigenous Australians are primarily driven by preventable infectious and chronic diseases [12,13,14]. These health inequalities are not limited to poverty only but are also influenced by broader social determinants of health, such as education, training, and skills development; employment status; access to and improvement in health care systems including technological innovation; transportation and food; conditions of homes and workplaces; social support; and gender and ethnicity [15,16]. Therefore, in addition to solely addressing biomedical challenges, it is imperative to reduce structural inequities in society through a more equitable distribution of community infrastructure resources, income, goods, and services for the holistic health and wellbeing of its people [15,17,18]. Many of these social determinants are fundamental to health and are associated with Indigenous people’s health and wellbeing [19,20]. There is concern that financial investments in Indigenous health are focused on behavior change rather than changing the environment and are not leading to improvements in health indicators [21]. 

Commonwealth, state, and territory governments in Australia fund strategic programs and services to improve the living conditions in Indigenous communities to reduce the disparities in health outcomes. As part of the “Closing the Gap” strategy, all levels of government use a range of indicators to monitor and evaluate the performance and functioning of essential services and community infrastructure. These indicators provide insight into population-related health and wellbeing and help set priorities for program and service delivery within the health and social service system [22,23]. Addressing the community determinants of health and wellbeing may improve community health and reduce the financial burden on the health care system.

Despite an emphasis on evidence-informed policy and accountability [24], the basis for the selection of indicators by government to monitor improvements in Indigenous health and wellbeing and the local relevance of these indicators is not always clear [25,26]. A system-wide approach to identify indicators for transparent priority setting and engagement with Indigenous community stakeholders is also lacking [24]. To generate policy- and practice-relevant evidence, a scoping review was conducted to identify community-informed built and socio-political environmental factors relevant to remote Indigenous community health and wellbeing, inclusive of infectious and chronic diseases. A scoping review methodology was chosen as it allows key concepts and evidence from diverse sources to be summarized and consolidated to inform policy and practice and guide future research [27]. Scoping reviews often include stakeholder consultation to validate research findings [28] and make recommendations for future research [29]. Although scoping reviews have been adopted in various disciplines and fields to answer a range of research questions [27,30], a recent review found that out of 344 scoping reviews, 58.7% addressed a health-related topic [31]. Most of these reviews rely on peer reviewed literature sources [32,33]. This scoping review study is novel for systematically reviewing publicly available grey literature [34]. The grey literature pertains to a range of print and electronic documents produced by government, not-for-profit, academic, business, and industry sources that are not controlled by commercial publishers [34]. This scoping review utilized policy and planning-related documents to achieve the following research objectives:

1. identify and characterize the key characteristics of publicly available planning, policy, and reporting documents relevant to remote Indigenous communities in the NT;
2. classify community-level built and socio-political environmental indicators using an Indigenous indicator classification system (IICS).

## 2. Study Context

This scoping review was supported by an Australian National Health and Medical Research Council (NHMRC) funded project grant titled Environments and Remote Indigenous Cardiometabolic Health (EnRICH). The EnRICH Project aimed to characterize remote Indigenous communities in the NT according to their social, built, and physical environmental characteristics in relation to community-level cardiometabolic disease outcomes. This scoping review used the initial pool of EnRICH-identified 51 remote communities of the NT as the basis for its study sample. 

This review applied an integrated knowledge translation (iKT) approach [35,36]. Decision makers, including policy officers, senior administrators, and members of the public and environmental health workforce in the NT were engaged throughout the scoping review process. These workforce members were considered to have expert knowledge on local living conditions based on their experiences living and working in remote Indigenous communities in the NT. The stakeholder organizations identified a need to investigate how community-level environmental living conditions related to both infectious and chronic diseases, rather than focusing solely on chronic disease. The review responded to identified stakeholder concerns and was expanded to include infectious disease. Discussions with stakeholders then lead to a re-framing of the review to health and wellbeing. From an Indigenous perspective, “health and wellbeing” is inclusive of chronic and infectious diseases outcomes [37]. 

To ensure cultural integrity of the study, two Indigenous cultural mentors were engaged. Indigenous cultural mentors provided cultural oversight at the early stage in framing the scoping review protocol and in subsequent stages of the review process. The stated aim of this scoping review reflects the input from stakeholder organizations and Indigenous cultural mentors.

## 3. Materials and Methods

This scoping review followed Arksey and O’Malley’s six-stage methodological framework [28] and Levac’s enhancement recommendations [38]. Each stage is identified below with the relevant enhancements. 

Stage 1: Identify research question 

Guided by a conceptual framework of Indigenous place and health [4], the scoping review addressed the following research question: *For Indigenous people living in remote communities in the Northern Territory, what local community-level built and socio-political environmental indicators are available and relevant to the health and wellbeing, inclusive of chronic disease and infectious disease, from Indigenous community members’ perspectives?*

Stage 2: Identify relevant studies (literature)

A robust search was undertaken to identify relevant grey literature documents published online. 

The final selection of documents was distilled from data sources, including Indigenous databases, which were most likely to house peer reviewed and non-peer reviewed literature relevant to Indigenous populations. Data sources were websites of local, regional, and state/territory governments; websites of community-controlled health organizations; and electronic databases of research repositories such as the Australian National Library (i.e., TROVE), and the Aboriginal and Torres Strait Islander Health Bibliography. The search was limited to literature published between 2001 and 2015. The following search terms were used to identify relevant literature:

- Indigenous Australian OR Aboriginal OR Torres Strait Islander AND;
- community AND;
- environment AND;
- plan OR policy OR report AND;
- Northern Territory OR NT.

Stakeholder organizations suggested the following document types as the starting point to identify community-level environmental indicators: (i) strategic plans, (ii) local implementation plans, (iii) community plans, (iv) corporate plans, (v) operational plans, (vi) annual reports, and (vii) evaluation reports. 

Stage 3: Study selection

A two-stage study selection process was applied. First, the following three criteria were used to identify organizations as data sources relevant to the review question:

- The organization is responsible for the delivery of community health and environmental programs to Indigenous people specific to one or more remote communities in the NT;
- the organization publishes community-level policy, reporting, evaluation, strategic planning, or local planning documents with a focus relevant to health and wellbeing, including preventable infectious disease and chronic disease; and
- the organization is a member of a peak body in the NT.

To be eligible for selection, the organizations needed to satisfy all three criteria.

Second, the documents were selected based on a set of six inclusion criteria and decision rules (see Table 1). All six criteria were required to be met for a document to be included in the review.

Stage 4: Charting the data

A “data charting tool” was used to extract information (data) from the included planning documents. It comprised two distinct components: (1) extraction of descriptive information (e.g., title, source, year published), and (2) extraction and classification of indicators. The second component integrated a culturally-relevant Indigenous indicator classification system (IICS) [39]. Indicators were classified according to its four hierarchical levels: (i) Subject Grouping, (ii) Domain, (iii) Goal Dimension, and (iv) Indicator Group. Each of the charted indicators was assigned to a numeric sub-dimension number (1, 2, 3, etc.) for identification purposes. 

Stage 5: Collating, summarizing, and reporting the results 

Extracted data were collated and analyzed using SPSS [40]. Descriptive statistics, i.e., percentage, mean, median, and interquartile range were computed, as appropriate. The analyses were stratified according to: regional area (i.e., Northern, Central, Big Rivers); IICS classification level (i.e., subject group, domain, goal dimension, indicator group); level of governance (i.e., local, regional); and document type (i.e., local implementation plan, community plan, local authority plan, regional plan).

Stage 6: Consultation exercise

This study engaged Indigenous and non-Indigenous frontline workers, as well as managerial and policy-level staff members, who worked in, or had responsibility to provide, environmental and/or public health services in one or more of the identified communities in the NT. The engagement of these stakeholders in a working group meeting setting was key to consolidating and synthesizing the built environmental indicators generated from the scoping review. 

## 4. Results

**Figure 1:**
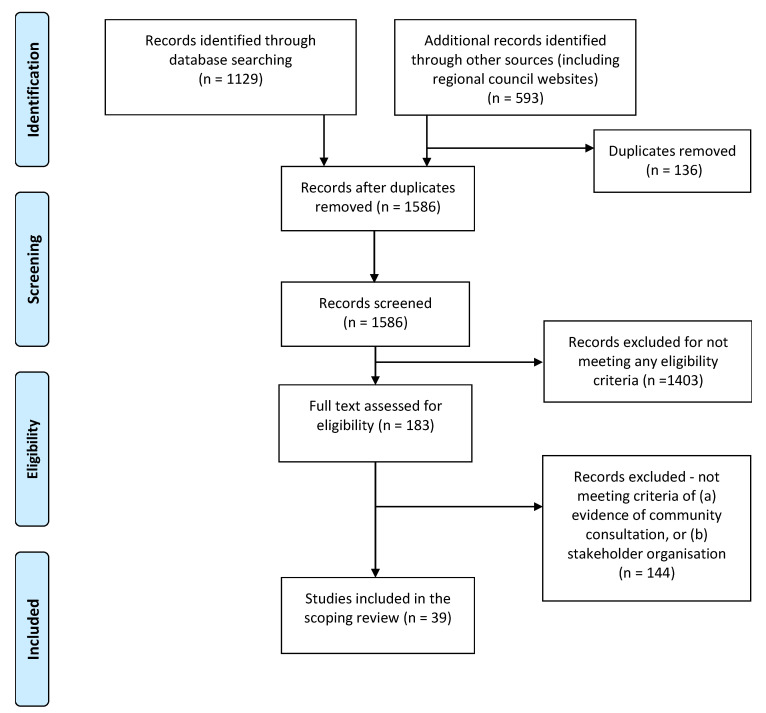
Process of identifying and selecting primary documents.

The planning documents were distilled from an initial pool of 1722 records (through database searching and other sources) that included both academic journal articles and grey literature documents. After deduplication, 1586 records were screened and assessed against the eligibility criteria. The eligibility assessment identified 39 planning documents for inclusion in the review (see Figure 1). 

Of the included documents, 31 were community-level plans classified as local implementation plans (*n* = 13), community plans (*n* = 10), and local authority plans (*n* = 8). The remaining eight documents were community-focused regional-level plans classified as regional plans. The timeframes for the documents ranged from 1 to 3 years (median = 1.0, interquartile range 1.0, 3.0) for the community-level plans, and 1 to 4 years (median = 1.0, interquartile range 1.0, 1.0) for the regional-level plans. The number of pages ranged from 1 to 62 (median = 27.5, interquartile range 3.5, 52.0) for community-level plans, and from 31 to 147 (median = 81.5, interquartile range 42.3, 125.8) for regional-level plans. 

A total of 2481 indicator statements were identified from the included planning documents. Overall, the number of indicator statements per document ranged from 6 to 163 (median = 66.0, interquartile range 24.0, 99.0) statements. Approximately 77% (*n* = 1913/2481) of the indicator statements, were classified as relevant to the built or socio-political environment and were eligible for extraction. The remaining indicator statements (23%) were aligned with socio-economic, socio-demographic, and cultural environments that were outside the scope of this study. Table 2 shows the characteristics of selected indicator statements related to socio-political and built environments by type of planning document. Results are further presented according to hierarchical classification levels of the IICS (see Supplementary File, Table S1).

Subject Group level

On average, each indicator statement represented more than one element of the built and/or socio-political environment. Disaggregating the multiple elements from the initial pool of 1913 indicator statements resulted in 3335 distinct indicators. The majority of these distinct indicators were related to the socio-political environment subject group (66.4%); the remaining indicators were related to the built environment subject group (33.6%). 

Indicator statements within the socio-political subject group were more prominent than indicator statements within the built environment subject group for all three regions of the NT, namely Northern (73.3%, *n* = 1025), Big Rivers (64.0%, *n* = 529), and Central Australia (59.6%, *n* = 661); and for the document types of local implementation plans (73.1%, *n* = 1480) and regional plans (70.1%, *n* = 469). 

Domain Level

**Figure 2:**
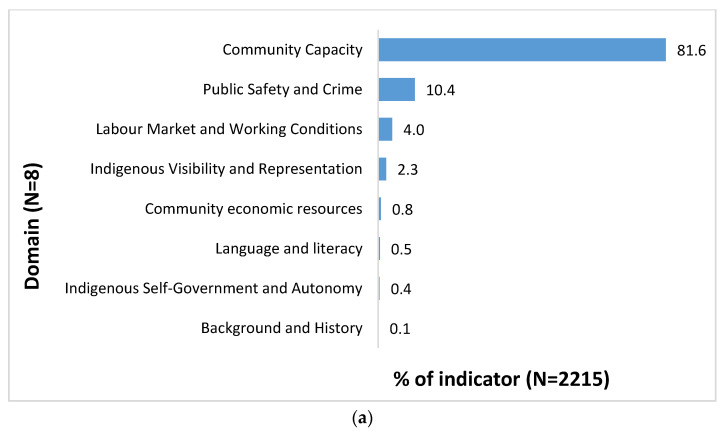

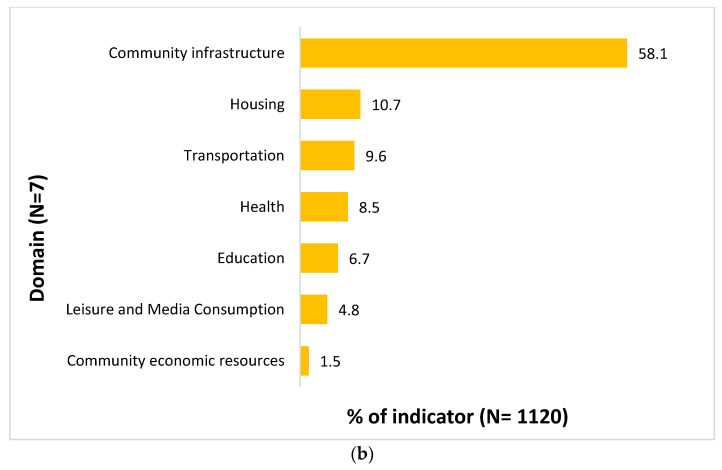
(**a**) Distribution of indicators by domain for the socio-political environmental subject group; (**b**) distribution of indicators by domain for the built environmental subject group.

The indicator statements were classified into 15 domains; 8 of these domains were aligned with the socio-political environment subject group and 7 domains with the built environment subject group, as illustrated in Figure 2. Within the socio-political subject group, the majority indicators (Figure 2a) were related to the *community capacity* (81.6%, *n* = 1808), followed by the *public safety and crime* (10.4%, *n* = 230), and *labor market and working conditions* (4.0%, *n* = 88) domains. Within the built subject group, the majority indicators (Figure 2b) were related to the *community infrastructure* (58.1%, *n* = 651), followed by the *housing* (10.7%, *n* = 120), *transportation* (9.6%, *n* = 108), *health* (8.5%, *n* = 95), and *education* (6.7%, *n* = 75) domains.

At the socio-political environmental domain level, components of community capacity include aspects of community governing structure and processes, and social and inter-organizational networks. Community capacity also involves opportunities for skills development training, community members’ abilities to participate in advocacy and mobilization, and community economic and social capital. The public safety and crime domain pertains to the availability of resources for community protection (e.g., legal protection) and emergency preparedness, and policies on public and environmental health practices. Labor market and working conditions reflect that within communities there are existing stable employment and reemployment opportunities available, and that those who are not employed are provided with social assistance support. 

At the built environmental domain level, components of community infrastructure represent local-level infrastructure resourcing and social programming. The housing domain involves all aspects of housing. The transportation domain consists of transport facilities (e.g., bus, bus stop, train station). The health domain includes all facilities related to health services. Finally, the education domain includes available education and community centers within the community. 

Goal Dimension Level

**Figure 3:**
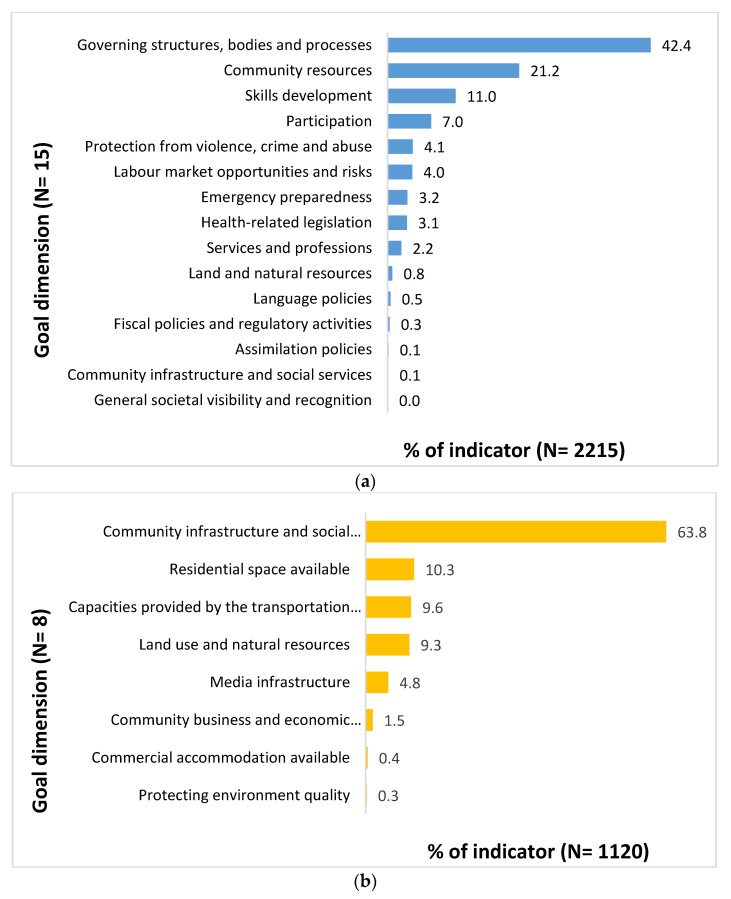
(**a**) Distribution of indicators by goal dimension for the socio-political environmental subject group; (**b**) distribution of indicators by goal dimension for the built environmental subject group.

The indicator statements were further classified into 23 goal dimensions; 15 of these goal dimensions were relevant to the socio-political environment subject group and 8 to the built environment subject group, as illustrated in Figure 3. The majority of socio-political indicators (Figure 3a) were classified to the goal dimensions of *governing structures, bodies and processes* (42.4%, *n* = 940), followed by *community resources* (21.2%, *n* = 470), *skills development* (11.0%, *n* = 243), and *participation* (7.0%, *n* = 155). The majority of built indicators (Figure 3b) were classified to the goal dimensions of *community infrastructure and social programming* (63.8%, *n* = 714), followed by *residential space available* (10.3%, *n* = 115), *capacities provided by the transportation system* (9.6%, *n* = 108), *land use and natural resources* (9.3%, *n* = 104), and *media infrastructure* (4.8%, *n* = 54).

At the socio-political environmental goal dimension level, the governing structures, bodies, and processes reflect opportunities and resources available for communities to establish their own governing bodies (e.g., a community elder’s council), the ability to undertake planning and policy development work, and the establishment and functioning of a network of organizations to support program and service delivery. The community resources goal dimension involves the establishment of community-based organizations for sustainable programs and service delivery, and the availability of program and organizational funding. The skills development goal dimension reflects a process whereby community members have the opportunity to improve their skills through training and education. Participation, on the other hand, involves community members’ engagement within internal community public affairs, engagement with external public affairs, and participation in the political arena. 

At the built environmental goal dimension level, community infrastructure and social programming consists of essential facilities and services that are required for community functioning. This includes facilities such as a community center, sports and recreational facilities, water and power supply infrastructure, and waste disposal facilities and services. The goal dimension pertaining to residential space available refers to spaces and infrastructure where people can live, specifically residential dwellings, common community dwellings (e.g., staff quarters), and incomplete residential building infrastructure. Capacities provided by the transportation system primarily involve existing transportation facilities including access and availability of buses, trains, and water transportation facilities complemented by a road network. The land use and natural resources goal dimension includes features of environments such as land development, vegetation, landscaping, and natural resources including sea, minerals, and waterbodies. The media infrastructure goal dimension represents facilities and services for communication including print and electronic media, telephone and mobile networks, and advanced community facilities such as internet. 

Indicator Group Level

**Figure 4:**
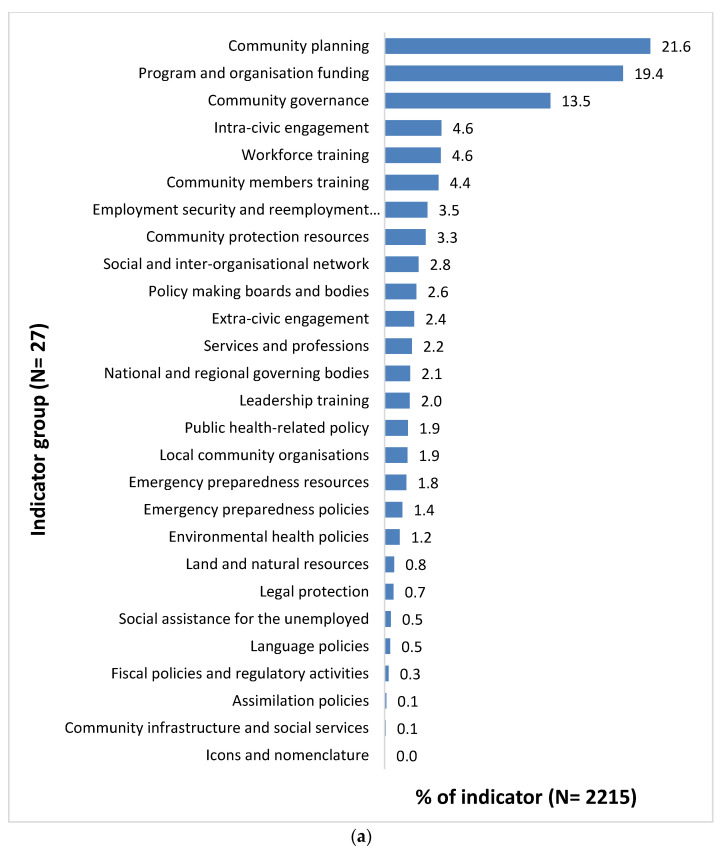

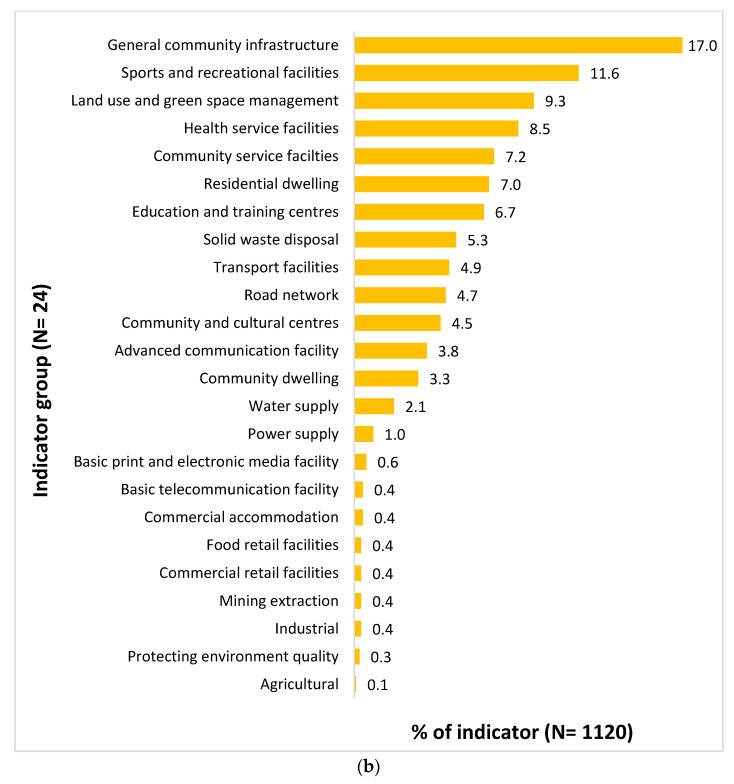
(**a**) Distribution of indicators by indicator group for the socio-political environmental subject group; (**b**) distribution of indicators by indicator groups for the built environmental subject group.

The indicators were then classified into 51 indicator groups, of which 27 were relevant to socio-political environments and 24 were relevant to built environments, as illustrated in Figure 4. The majority of socio-political environmental indicators (Figure 4a) were classified into the indicator groups of *community planning* (21.6%, *n* = 478), followed by *program and organization funding* (19.4%, *n* = 429), and *community governance* (13.5%, *n* = 298), while the majority of built environmental indicators (Figure 4b) were classified into the indicator groups of *general community infrastructure* (17.0%, *n* = 190), *sports and recreational facilities* (11.6%, *n* = 130), *land use and green space management* (9.3%, *n* = 104), *health services facilities* (8.5%, *n* = 95), *community service facilities* (7.2%, *n* = 81), and *residential dwelling* (7.0%, *n* = 78).

At the socio-political indicator group level, indicators related to community planning involved the community’s ability to undertake short- and long-term planning processes in diverse settings. These included a plan identifying the community priority for program and service delivery, for example, to develop an economic and opportunities profile. Indicators related to program and organizational funding refer to a broad range of programs and services implemented at the community-level and funding provided by federal, state, and regional governments. Indicators relevant to community governance often included establishment of groups or committees to oversee programs and services (e.g., establish a school attendance working group). 

At the built environmental indicator group level, general community infrastructure includes facilities and infrastructure that are not otherwise catalogued into relatively larger infrastructure (e.g., community hall), such as public toilets, shed, drainage and foot paths, cemetery, etc. Indicators related to sports and recreational facilities include spaces where people can participate in sports and leisure activities (e.g., ovals, basketball courts, swimming pool). Indicators related to land use and greenspace management comprise the development and management of parks and reserves, vegetation, furniture, and water fountains in parks. Indicators relevant to health services include facilities and services to provide primary and tertiary health care including community health center, aged care facility, pharmacy, hospital, morgue, and pest control/animal management. Community services facilities, on the other hand, include facilities such as laundry, garage/auto service, repairs and maintenance service, postal and financial services, government business center, and council office, whereas residential dwelling comprises residential housing facilities including free standing homes, units, and apartments. 

## 5. Discussion

This scoping review aimed to capture built and socio-political community-level environmental indicators relevant to the health and wellbeing of remote Indigenous communities in the NT Australia. The review was guided by an iKT approach with environmental and public health stakeholder organizations in the NT shaping the review scope and suggesting the use of community and regional planning documents developed through community inputs as a way forward for the review. This scoping review provides an illustrative example for utilizing grey literature planning documents to inform policy and practice and to guide future research. 

The review found that a greater proportion of indicators in the planning documents were related to the socio-political environment. This finding may reflect communities’ higher perceived needs related to the influence of local socio-political environments on community health and wellbeing, than the influence of built environments. 

Within the socio-political environment, indicators related to the community capacity domain were most prominent and reflected community governing structure and processes, as well as social and inter-organizational networks. This domain also features opportunities for skills development training, community members’ abilities to participate in advocacy and mobilization, and economic and social capital—conceivably pre-requisites for Indigenous community self-determination. Prominence of the community capacity domain supports Indigenous peoples’ right to make decisions on issues that affect them [41,42]. In a changing policy landscape, and to ensure application of effective use of scarce resources in servicing Indigenous communities, it is essential that individual, community, and organizational capacity is strengthened to enable priority setting and appropriate responses to local needs [42,43,44]. 

Within the built environment, indicators related to community infrastructure were featured prominently in the review. Community infrastructure generally consists of essential facilities such as the following: community center, sports and recreational facilities, water and power supply infrastructure, waste disposal facilities, and services that are required for adequate community functioning. These essential facilities are relevant to the health and wellbeing of remote Indigenous communities. For example, community infrastructure relevant to residential housing has long been associated with health and wellbeing, inclusive of infectious and chronic diseases in remote Australian Indigenous communities [45,46,47]. Non-housing related features of community infrastructure such as sports and recreational facilities can also impact infectious and chronic diseases through direct and indirect (mediational) pathways [3,4]. However, the non-housing-related features identified in this review have rarely been empirically assessed in the remote Indigenous Australian context. Most studies of this nature have been conducted in non-Indigenous and non-remote contexts [48].

Although the overall proportion of indicators related to the built environment found in the review was lower than the proportion of socio-political indicators, this does not imply that perceived community needs for built environments were less important. It may be that features of community-level built environments were adequate, or comparatively better addressed, than the community’s socio-political circumstances preceding the planning period. 

Interface between community capacity and community infrastructure

Community capacity, as an aspect of the socio-political environment, and community infrastructure, as an aspect of the built environment, complement each other in their influence on collective health and wellbeing. For example, communities may continue to grapple with the burden of infectious and chronic diseases if features of “community infrastructure” such as inadequate quality housing (e.g., non-functional sanitation and hygiene facilities), and features of “community capacity” such as unavailability of ongoing housing repairs and maintenance services persist [46,47]. This evidence is supported by the notion that the availability of community health infrastructure, and community capacity to manage such infrastructure, can impact health service delivery and program sustainability [49]. Further, the success and sustainability of a community-based health center, for instance, often depends on its capacity to develop inter-organizational networks to continue service delivery once funding ceases. Likewise, opportunities for social engagement within public spaces (e.g., cultural centers, churches, recreational facilities) can influence community members’ capacity to build networks and advocate for improved community-level built infrastructures [48]. 

Nevertheless, caution must be taken when considering the interplay between community capacity and community infrastructure, and their influence on health and wellbeing. An expectation that the community should solve their own problems by doing something about deficient community infrastructure, without adequate support and resourcing, is tantamount to “victim-blaming” and can reinforce disempowerment [50,51]. Health outcomes may vary between and within communities not only due to favorable existing health hardware, but also due to multifaceted factors including continuation of organizational funding, a stable workforce, and skilled local community members. Such variations in health outcomes could in turn influence the variations in existing community capacity and community infrastructure that require closer scrutiny.

Differences according to geographic region and planning document type

The distribution of built and socio-political environmental indicators in the documents varied according to the three regions of the NT. This finding suggests differences in the underlying social dynamics and infrastructure needs in communities within the regions. The socio-political environmental indicators frequently endorsed for the Northern region highlights communities’ greater aspirations for participation in governance, control of community resources, and strengthening skills to self-manage community-level built infrastructure. Therefore, needs for community governance and control, and strengthening skills to self-manage infrastructures in Northern region are likely to be greater than the Central and Big Rivers regions. In contrast, the built indicators frequently endorsed for the Central Australia region may reflect greater built infrastructure needs for Central Australia compared to the Northern and Big Rivers regions. This may be due to climatic conditions that will likely require new design specifications for community infrastructure (e.g., improved insulation and household air conditioning, stable electricity poles) to match localized extreme weather patterns in Central Australia [52]. 

Our findings show the distribution of socio-political environmental indicators were more prominent within the local implementation plan and regional plan documents, whereas the distribution of built environmental indicators were relatively higher in the community plan document compared to other document types. This difference may suggest diversity in the planning focus, and the roles and responsibilities involved in the establishment and maintenance of local level infrastructure. For example, typically regional-level planning documents have a strategic focus with a longer-term duration (e.g., 4 years), covering large areas and numbers of communities that fall within the structure of a regional government council. This is in contrast to community-level planning documents, which are based on short term (e.g., 1–2 years) local-level priority setting that complements regional-level strategic plans [53]. Community-level planning documents generally guide and inform a regional council’s service delivery responsibilities, including budgetary allocations towards its communities, on a year-by-year basis. Conversely, regional plans are used as a strategic framework to identify community-level needs and work out detail implementation over time. 

Utility of grey literature use 

A novel aspect of this scoping review was the use of grey literature, specifically planning documents. There is a growing recognition of the importance of grey literature to inform evidence-based decision making [54]. Although the methodologies used in grey literature may not be as robust as those appearing in peer-reviewed journals, grey literature is produced by practitioners, service providers, and community members who are local knowledge holders [54,55]. Grey literature often provides useful information relevant to applied settings such as assessment and innovation in public health practice on what place-based interventions are effective, why, and for whom [56,57]. Moreover, grey literature encapsulates policy and planning documents that are outside the realm of research and are generated by different levels of government to guide policy, practice, and service delivery. Relevant community and regional-level planning documents pertaining to built and socio-political environments were therefore thought to primarily be available in a broad range of grey literature information sources. 

The key findings of this study must be interpreted in the context of a number of strengths and limitations. 

Strengths

This study had three notable strengths. First, a key strength and unique characteristic of this scoping review was its use of the culturally-relevant IICS [39,58]. The IICS was adapted to guide the classification of environmental indicators as “perceived needs” of the community in a systematic approach. The structured framework of the IICS also enabled indicators classified at different hierarchical levels to be explored by region, which may be helpful in understanding differences in indicator distribution due to contextual differences within the regions. 

Second, Indigenous communities are over-researched and burdened with consultations by external stakeholder organizations soliciting the same type of information, and yet tangible benefits to the community from the research are often absent [59,60]. This scoping review explicitly prioritized privileging and acknowledging community perspectives in the selection, extraction, and classification of indicators by using existing documents developed with community consultation, to reduce community response burden. This approach provided a more compelling platform to engage key stakeholder organizations in interpreting the results.

Third, this study was additionally novel for informing two subsequent practice- and policy-relevant investigations. The synthesized built environmental indicators distilled from the scoping review were further refined by the working group and informed a second concepting mapping study to prioritize built environment indicators in relation to their influence on infectious and chronic diseases in the same remote region. Highly ranked and prioritized built environment indicators were then selected for a third epidemiological study examining associations between the built environment and infectious diseases in remote NT communities.

Limitations 

This study also had two notable limitations. First, it was a time-bound study. Due to time limitations, it was not possible to expand the search to include more than the 100 hits in Google. Presumably, the top 100 hits from the worldwide web were the most topic-relevant. Given the limited peer-reviewed literature on the socio-political and built environment in relation to the health and wellbeing of Indigenous communities in developed countries, including Australia [58], it is likely that few published studies relating to remote and Indigenous communities in the NT would have been identified. Therefore, this project relied on grey literature documents available through relevant stakeholder organizations and community websites. The list of included documents was reviewed by key government stakeholders and deemed to reflect relevant planning documents for these regions and communities. However, it is possible that some key documents may have been missed in the search process.

Second, although “evidence of community consultation” was one of the most important document selection criteria, it is possible that some documents were included that reflected tokenistic participation, and that some documents were excluded due to poor reporting in relation to level of community consultations. The reviewed documents could have been more explicit in how community members were involved in the planning process. Specifically, as suggested by Kenny et al. [61], more information could have been provided on how the community consultations were done, the nature of the outputs, and how the community was represented.

## 6. Conclusions

This review was an innovative first effort to identify and classify social-political and built environment indicators from community-level planning documents (“grey literature”) relevant to remote Indigenous communities in the NT, Australia. This review demonstrated the utility of adopting an iKT approach to respond to stakeholder organizations’ issues and concerns. To privilege community member perspectives and to avoid burdening community members with requests for information that they had already provided, this study included documents in the review that demonstrated evidence of community engagement. The use of a structured culturally-relevant indicator classification system (i.e., the IICS) was advantageous for systematically classifying the indicators. 

The embodiment of a classification system, such as the IICS, into public and environmental health practice may increase the scope for governments to coordinate service delivery in a uniform and consistent manner and could be used to inform priority setting for resource allocation as well as monitoring progress in service delivery in the NT, Australia. Future research should utilize participatory (action) research and the IICS to guide the structure and format of community-level planning processes. This would ensure greater consistency between planning and reporting documents and facilitate the comparison of performance monitoring based on indicator groups and domains. There is a growing call by population and public health researchers that future research on the disparities in Indigenous health should target community-level social environments as units of analysis [4,62]. The findings of this scoping review, specifically that 66% of extracted indicators were classified into the socio-political environment subject grouping compared to the built environment subject grouping, support this research focus.

From a policy and practice perspective, to maintain a healthful community living environment, it is important for communities to have access to supportive social and economic environments. Components of supportive environments may include adequate levels of income and employment opportunities and participation of the community in local decision making. Findings from this scoping review support this need. The review identified that the most common socio-political environmental indicators relevant to “community capacity” were “community control in planning and governance”, and “opportunities for employment, education, and training”. Therefore, systematically selected built and social environmental indicators generated in the scoping review, may support stakeholders from the environmental and public health sectors to identify priority indicators that can be actioned in the shorter and longer term. The built environmental indicators identified in this research may also contribute to a stronger evidence base to address local-level environmental health issues and strengthen the capacity of public and environmental health practice in the NT. 

## Figures and Tables

**Table 1:** Inclusion criteria used for identification and selection of relevant studies.

Criterion No.	Descriptions
1.	Specific to Indigenous people living in one or more remote communities identified for the EnRICH Project in the NT, Australia.
2.	Represent policy, reporting, evaluation, strategic planning, or local planning documents with a focus relevant to health and wellbeing, including preventable chronic disease and/or infectious disease.
3.	Must contain one or more identifiable community-level objective or subjective built and socio-political environmental indicators relevant to the social determinants of health.
4.	Show evidence of consultation (e.g., provide details on who participated, venue, date, and nature of input provided and not merely refer to “the community was consulted”) with Indigenous community members, representatives, or frontline professionals working in the areas of public and environmental health.^1^
5.	Publicly available online or in other formats published by government and non-government organizations in the most recent year but not earlier than 2001.
6.	Regional and state or territory level documents were included if they had relevance to one or more communities identified for the EnRICH Project in the NT, Australia.

^1^ Instances where insufficient detail of the community consultation was provided in a document, the first author investigated the source organization’s website for evidence of community consultation pertaining to that document (e.g., regional council and local authority meeting agenda and minutes where participants have workshopped for community priorities and discussed draft regional plans with stakeholders; photos of consultations).

**Table 2:** Descriptive characteristics of unique indicator statements related to socio-political and built environments according to type of planning document.

Types of Documents	Descriptive Statistics	No. of Unique Indicator Statements	
		Included	Excluded
Community-level plan (*n* = 31)	N (number of indicator statements)	1458	276
	Mean	47.0	8.9
	SD	35.1	7.3
	Median	46.0	8.0
	1st Qtl (0.25)	16.0	2.0
	3rd Qtl (0.75)	72.5	13.0
	Min	5	0
	Max	120	30
Regional-level plan (*n* = 8)	N (number of indicator statements)	455	265
	Mean	56.9	33.1
	SD	33.0	22.8
	Median	58.5	42.0
	1st Qtl (0.25)	32.7	11.5
	3rd Qtl (0.75)	79.25	47.0
	Min	9	3
	Max	99	62

## Supplementary Materials

The following are available online at https://www.mdpi.com/article/10.3390/ijerph18084167/s1, Table S1: Built and socio-political environmental indicator classification framework: title: Identifying environmental determinants relevant to health and wellbeing in remote Australian Indigenous communities: a scoping review of grey literature.
