# Supplementary material for: Identifying Environmental Determinants Relevant to Health and Wellbeing in Remote Australian Indigenous Communities: A Scoping Review of Grey Literature
Source: Int J Environ Res Public Health. 2021 Apr 15;18(8):4167. doi: 10.3390/ijerph18084167 (PMC8071139; doi:10.3390/ijerph18084167)
Supplement: Supplementary file 1 [file ijerph-18-04167-s001.zip › Chakraborty_scoping review_suppl file_table s1_submitted.pdf]

Supplementary File

Title: Identifying environmental determinants relevant to health and wellbeing in remote Australian Indigenous communities: a scoping review of grey literature

Authors: Chakraborty, A.; Daniel, M.; Howard, NJ.; Chong, A.; Slavin, N.; Brown, A.; and Cargo, M.

Table S1: Built and Socio-political environmental indicator classification framework.

Indicator ID	Indicator Group	Goal Dimension	Domain	Subject Group
4	Agricultural	Community business and economic development	Community economic resources	Built
9	Commercial accommodation	Commercial accommodation available	Housing	Built
10	Commercial retail facilities	Community business and economic development	Community economic resources	Built
11	Community and cultural centres	Community infrastructure and social programming	Community infrastructure	Built
12	Community dwelling	Residential space available	Housing	Built
19	Community service facilities	Community infrastructure and social programming	Community infrastructure	Built
20	Education and training centres	Community infrastructure and social programming	Education	Built
31	Food retail facilities	Community business and economic development	Community economic resources	Built
32	General community infrastructure	Community infrastructure and social programming	Community infrastructure	Built
33	Health service facilities	Community infrastructure and social programming	Health	Built

Indicator ID	Indicator Group	Goal Dimension	Domain	Subject Group
37	Incomplete building	Residential space available	Housing	Built
39	Industrial	Community business and economic development	Community economic resources	Built
43	Land use and green space management	Land and natural resources	Community infrastructure	Built
49	Mining extraction	Community business and economic development	Community economic resources	Built
54	Power supply	Community infrastructure and social programming	Community infrastructure	Built
62	Residential dwelling	Residential space available	Housing	Built
71	Solid waste disposal	Community infrastructure and social programming	Community infrastructure	Built
72	Sports and recreational facilities	Community infrastructure and social programming	Community infrastructure	Built
78	Water supply	Community infrastructure and social programming	Community infrastructure	Built
2	Access to working place	Capacities provided by the transportation system	Transportation	Built
3	Advanced communication facility	Media infrastructure	Leisure and Media Consumption	Built
7	Basic print and electronic media facility	Media infrastructure	Leisure and Media Consumption	Built
8	Basic telecommunication facility	Media infrastructure	Leisure and Media Consumption	Built
25	Environmentally damaging activities	Environmentally damaging activities	Community infrastructure	Built
51	Negative social impact	Negative impact of the transportation system	Transportation	Built
57	Protecting environment quality	Protecting environment quality	Community infrastructure	Built
58	Provision of environmental goods	Provision of environmental goods	Community infrastructure	Built
60	Quality of housing unit amenities	Quality of housing unit amenities	Housing	Built
64	Road network	Capacities provided by the transportation system	Transportation	Built

Indicator ID	Indicator Group	Goal Dimension	Domain	Subject Group
75	Transport facilities	Capacities provided by the transportation system	Transportation	Built
76	Travel purposes	Capacities provided by the transportation system	Transportation	Built
77	Volume of traffic	Capacities provided by the transportation system	Transportation	Built
5	Assimilation policies	Assimilation policies	Background and History	Socio-political
6	Awards, monuments and holidays	General societal visibility and recognition	Indigenous Visibility and Representation	Socio-political
14	Community governance	Governing structures, bodies and processes	Community Capacity	Socio-political
15	Community infrastructure and social services	Community infrastructure and social services	Indigenous Self-Government and Autonomy	Socio-political
16	Community members training	Skills development	Community Capacity	Socio-political
17	Community planning	Governing structures, bodies and processes	Community Capacity	Socio-political
18	Community protection resources	Protection from violence, crime and abuse	Public Safety and Crime	Socio-political
21	Emergency preparedness policies	Emergency preparedness	Public Safety and Crime	Socio-political
22	Emergency preparedness resources	Emergency preparedness	Public Safety and Crime	Socio-political
23	Employment security and reemployment opportunities	Labour market opportunities and risks	Labour Market and Working Conditions	Socio-political
24	Environmental health policies	Health-related legislation	Public Safety and Crime	Socio-political
26	External recognition of homelands or traditional lands	Reconciliation mechanisms or events	Background and History	Socio-political
27	Extra-civic engagement	Participation	Community Capacity	Socio-political
29	Family separation	Family separation	Background and History	Socio-political

Indicator ID	Indicator Group	Goal Dimension	Domain	Subject Group
30	Fiscal policies and regulatory activities	Fiscal policies and regulatory activities	Indigenous Self-Government and Autonomy	Socio-political
34	Historical events	Historical events	Background and History	Socio-political
35	Human development	Human development	Social Welfare	Socio-political
36	Icons and nomenclature	General societal visibility and recognition	Indigenous Visibility and Representation	Socio-political
40	Intra-civic engagement	Participation	Community Capacity	Socio-political
42	Land and natural resources	Land and natural resources	Community Economic Resources	Socio-political
44	Language policies	Language policies	Language and literacy	Socio-political
45	Leadership training	Skills development	Community Capacity	Socio-political
46	Legal protection	Protection from violence, crime and abuse	Public Safety and Crime	Socio-political
47	Local community organisations	Community resources	Community Capacity	Socio-political
50	National and regional governing bodies	Governing structures, bodies and processes	Community Capacity	Socio-political
52	Participation in the political context	Participation	Community Capacity	Socio-political
53	Policy making boards and bodies	Governing structures, bodies and processes	Community Capacity	Socio-political
55	Program and organisation funding	Community resources	Community Capacity	Socio-political
56	Programs for Indigenous community development	Programs for Indigenous community development	Indigenous Visibility and Representation	Socio-political
59	Public health-related policy	Health-related legislation	Public Safety and Crime	Socio-political
61	Recognition of other losses	Reconciliation mechanisms or events	Background and History	Socio-political
63	Return or recognition of removed artefacts	Reconciliation mechanisms or events	Background and History	Socio-political
65	Services and professions	Services and professions	Indigenous Visibility and Representation	Socio-political
66	Settled claims	Settled claims	Community Economic Resources	Socio-political

Indicator ID	Indicator Group	Goal Dimension	Domain	Subject Group
67	Social and inter-organisational network	Governing structures, bodies and processes	Community Capacity	Socio-political
68	Social assistance for the unemployed	Labour market opportunities and risks	Labour Market and Working Conditions	Socio-political
69	Social dependence	Social dependence	Social Welfare	Socio-political
70	Social expenditures	Expenditure for the transportation system	Transportation	Socio-political
73	State affiliation and identification	State affiliation and identification	Background and History	Socio-political
79	Workforce training	Skills development	Community Capacity	Socio-political
